# Therapeutic Upper Gastrointestinal Endoscopy in Pediatric Gastroenterology

**DOI:** 10.3389/fped.2021.715912

**Published:** 2022-02-25

**Authors:** Dominique Schluckebier, Nadeem Ahmad Afzal, Mike Thomson

**Affiliations:** ^1^Pediatric Gastroenterology, Sheffield Children's NHS Foundation Trust, Western Bank, Sheffield, United Kingdom; ^2^Department of Paediatrics, Southampton Children's Hospital, Southampton, United Kingdom

**Keywords:** endoscopy, therapeutic, pediatric, emergency, bleeding, varices, foreign body (FB), reflux

## Abstract

This paper seeks to give a broad overview of pediatric upper gastrointestinal (GI) pathologies that we are now able to treat endoscopically, acquired or congenital, and we hope this delivers the reader an impression of what is increasingly available to pediatric endoscopists and their patients.

## Introduction

The last 50 years has witnessed an explosion in what is therapeutically feasible *via* an endoscope in the gastrointestinal (GI) tract. This paper seeks to give a broad brushstroke of pediatric upper GI pathologies that we are now able to treat endoscopically, acquired or congenital and we hope this delivers the reader a taste of what is increasingly available to pediatric endoscopists and their patients.

## Emergencies in Upper GI Endoscopy

### Upper GI Bleeding (UGIB)

In the case of UGIB, endoscopy is often the intervention of choice as it is both diagnostic and therapeutic (1). However, procedures might require advanced endoscopy skills for efficient hemostasis and should therefore only be undertaken by experienced endoscopists, who have the ability to perform therapeutic procedures. This was true prior to the advent of topical hemostatic substances—but more of that later. Emergency endoscopy should not be realized in a hemodynamically unstable child and preferably performed after complete resuscitation within 12 h of admission in the case of variceal bleeding and within 24 h for non-variceal bleeding. If endoscopy is performed in the first 24 h after onset of symptoms, the chance to detect a bleeding lesion is over 80% but decreases significantly to <40% if performed after 48 h (1).

In adults, well-validated and robust scoring systems, like Rockall, Blatchford, and Forrest, have revolutionized the endoscopy intervention in UGIB (2). Based on parameters such as urea level, age, presence of comorbidities, and presence of “shock, ” these scoring systems identify not only patients at high risk (of repeat bleeding, need for blood transfusion, surgical intervention, and mortality), who require immediate endoscopic intervention, but also those patients of low risk, helping to avoid unnecessary endoscopies and interventions. These scoring systems are unfortunately not applicable in the pediatric population, as its hematological, biochemical, and physiological parameters differ from those of adults, with different values between age groups. Thomson et al. developed a scoring system to predict the need of endoscopic hemostatic intervention. It includes a total score of 24, involving history, clinical assessment, laboratory findings, and management and resuscitation, with a cutoff for intervention at 8 (Table 1). This “Sheffield scoring system” had a positive predictive value (PPV) of 91.18%, a negative predictive value (NPV) of 88.57%, and a sensitivity and specificity of 88.7 and 91.18%, respectively (2). Such scoring systems are extremely useful in identifying which child should receive potentially life-saving endoscopic hemostatic treatment.

**Table 1:** Sheffield Scoring System.

**History taking**
Significant pre-existing condition: 1
Presence of melaena: 1
History of large amount of hematemesis: 1
**Clinical assessment**
HR > 20 (from mean HR for age): 1
Prolonged CRT: 4
**Laboratory findings**
Hb drop > 20 g/L : 3
**Management and resuscitation**
Need for fluid bolus: 3
Need for blood transfusion (Hb <80 g/L): 6
Need for other blood product: 4
**Total score: 24**
**Cut-off: 8 (>** **8 considered as threshold for intervention)**

#### Variceal Bleeding

##### Esophageal Varices (EVs)

In advanced liver disease or portal vein thrombosis leading to portal hypertension, EVs are a common finding in children. Fortunately, EV rupture and associated mortality are rare in children, compared with the adult population, but may result in significant bleeding and represent a life-threatening condition which requires emergent endoscopic evaluation (3). The aim of endoscopic intervention is not only the cessation of EV bleeding but also the reduction of the variceal wall tension (by obliterating the varix), to prevent further bleeding episodes (4). However, treatment of variceal bleeding remains a challenging intervention even for experienced pediatric endoscopists, with potentially high complication rates, as described in the King's College Hospital report, with a complication rate of 37%, using banding, sclerosants (76%), or both in their study population (5). Complications include esophageal ulcers, esophageal strictures, and erosive gastritis. To date, there have been various techniques for EV treatment, which are discussed below.

**Figure 1:**
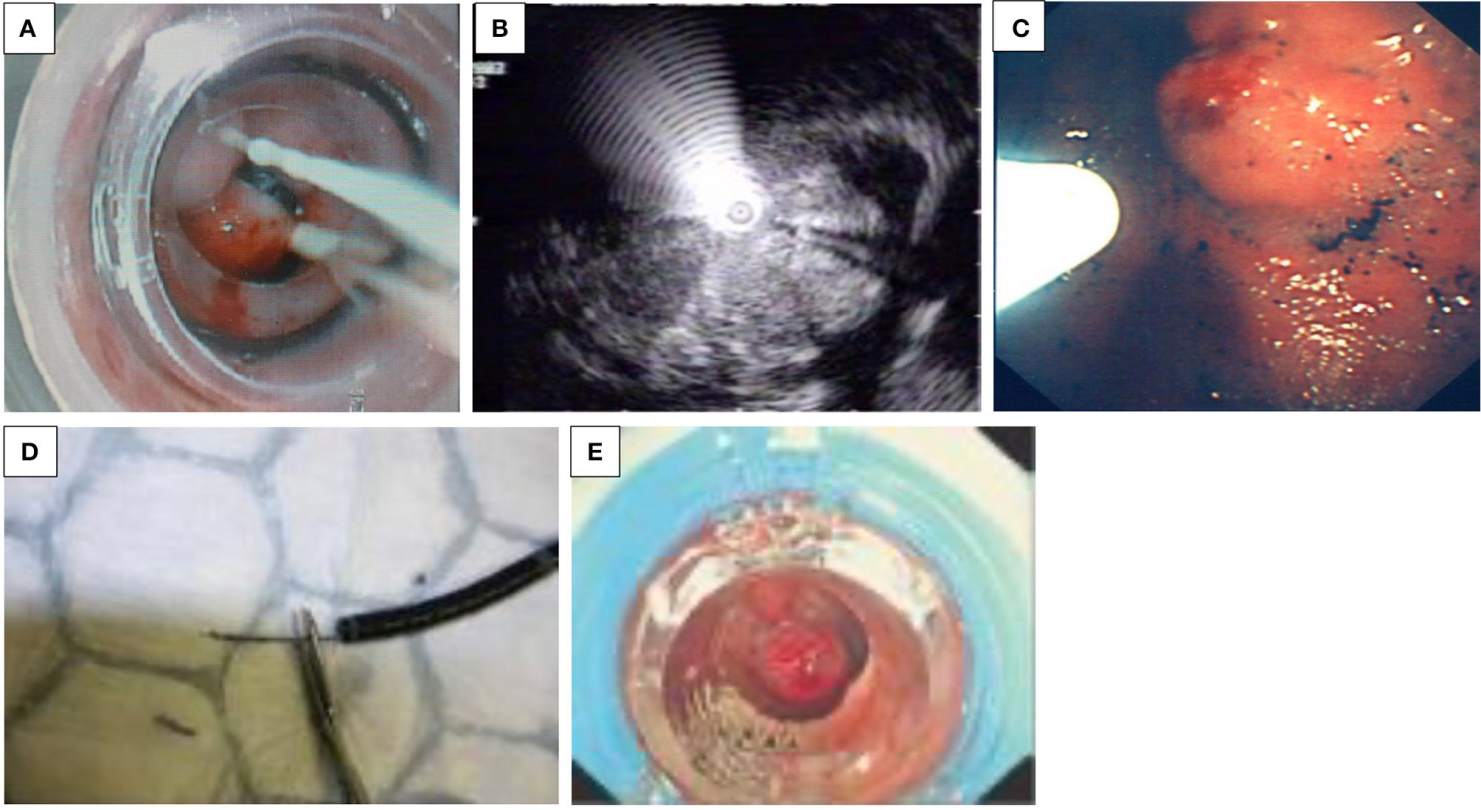
**(A–E)** Endoscopic treatment of variceal bleeding. **(A)** Banding of esophageal varices. **(B)** Echo-endoscopic vision of needle injection of an esophageal varix. **(C)** Injection of glue into a fundal varix. **(D)** An inadvertent introduction of glue into the biopsy channel is prevented by cutting the catheter tip off. **(E)** Banding of jejunal varix.

*Banding*. This is the first choice for EV bleeding, as meta-analyses have shown it to be superior to sclerotherapy in terms of higher eradication rates and lower rates of rebleeding and complications (6). Banding consists of the placement of rubber rings on the variceal column by sucking the varix into the plastic cylinder, attached to the tip of the endoscope (Figure 1A). In active bleeding, the focus should be on the point of bleeding, and inaccurate bands applied do not cause adverse events—in comparison to sclerotherapy (4). Originally, banding devices allowed the application of only one band at a time, which required reloading with each subsequent band ligation. Now, however, multiple band ligators may be applied at one intubation, which means that four to seven bands can be sequentially deployed without the need for repetitive intubating or the use of an overtube. These are manufactured for use with the adult-sized scopes and added with an extra 2–3 mm to the diameter, limiting its use in children younger than 12 months or under 8 kg.

The ligated tissue with the rubber band may fall off between 1 and 10 days after the procedure (4). It is therefore crucial to inform the family about the increased risk of bleeding recurrence during this time span. Repeat endoscopy before discharge is considered advisable in cases of acute bleeding.

*Sclerosants*. The use of variceal injection (Figure 1B) is less popular in the pediatric population and is only usually indicated in younger children, where banding is difficult due to the diameter of the scope exceeding the esophageal diameter—specifically the cricopharyngeal narrowing. Efficacy and complication rates vary among pediatric studies. Eradication varies between 11 and 87% in different studies (3, 7). Complications related to sclerotherapy are esophageal ulcers, strictures, and erosive gastritis (8).

Various sclerosing agents are available and can be classified as follows:

– Synthetic (sodium tetradecyl sulfate 1 and 3%, polidocanol 0.5–3%)
– Fatty acid derivatives (ethanolamine oleate 5%, sodium morrhuate 5%)
– Alcohol (ethanol 99.5%, phenol 3%)
– Sugars (hypertonic 50% dextrose solution).

Individual discussion of the pros and cons of these agents is beyond the scope of this article.

##### Gastric Varices

Injection of Histoacryl “glue” is the technique of choice, although complications such as fever, infection, gastric ulcer, damage to/blockage of the endoscope, perforation, and peritonitis can occur (9). There is a non-negligible risk of embolization of collateral vessels or other organs, in particular if an insufficient amount of cyanoacrylate has been injected (9). As in adults, embolization is a potential risk, with a higher risk for systemic embolization in case of the presence of a right-to-left intracardiac communication such as an atrial or ventricular septal defect (10).

*N*-Butyl-2-cyanoacrylate (*n*-BCA, NBCA) is an efficient injection substance in acute esophageal and gastric variceal bleeding and for obliteration of fundal varices (Figure 1C). In children, the use of the glue injection technique has been utilized in infants in whom the diameter of the esophagus may preclude introduction of the banding devices, and in pilot studies, it seems effective and safe in the short term, with a rebleeding rate of 3/8 young children under 2 years old within 12 weeks (11). The main complication was rebleeding resulting from extrusion; the prognosis of the patients depended on the severity of the underlying liver disease (12). Other side effects include glue extrusion and potential damage to the biopsy channel of the endoscope minimized in skilled hands. This certainly dictates that the operator employs great care when performing this procedure. As Figure 1D demonstrates, the ideal technique following glue delivery is to cut the catheter tip off with any extraneous glue attached before withdrawing through the biopsy channel, while maintaining suction to prevent inadvertent introduction of the glue into the biopsy channel. Thrombin may be used, and patients usually receive one to four sessions of thrombin, with a mean total dose of approximately 10 ml for variceal eradication (13).

##### Intestinal Varices

Recurrent UGIB due to intestinal varices is rare. Apart from portal hypertension, intestinal varices can develop after intestinal surgery, by accidental venous occlusion, by microthrombi, or by accidental ligature during surgery, leading to the development of collateral vessels. While they account for up to 5% of all variceal bleeding in adults with portal hypertension, to date, only a few case reports exist in the pediatric population. Belsha and Thomson reported an 8-year-old with jejunal varices with short-bowel syndrome after multiple surgeries for gastroschisis and duodenal and colonic atresia, which was successfully treated by banding (Figure 1E) (2).

An alternative is radiological coil stenting as surgical intervention would include the resection of the reanastomosis, which might be challenging due to adhesions secondary to repeated surgical interventions.

#### Non-variceal Gastric and Small-Bowel GI Bleeding

UGIB related to lesions in the stomach include, among other pathologies, diffuse hemorrhagic gastritis, Dieulafoy's lesions, other angiodysplasias, and peptic ulcer disease (PUD). The incidence of PUD in children is much lower than in the adult population, varying between 2 and 8% and between 0.5 and 4.4 of 100,000 individuals in case of UGIB (14). There are various treatment options for GI bleeding, including injection of sclerosing or hemostatic agents, thermocoagulation techniques, and different clip devices. Epinephrine (1:10,000–1:100,000) can be used in acute situations in order to identify the source, but it must be remembered that vasoconstriction and tamponade effects only last for 10–15 min, and it is therefore not to be used without more definitive subsequent therapy. Thermocoagulation techniques include monopolar and bipolar coagulation, argon plasma coagulation (APC), and laser photocoagulation. As these are well-described in textbooks, we will hence concentrate on more recent therapeutic developments (8).

##### Over-the-scope Clips (OTSC^®^)

**Figure 2:**
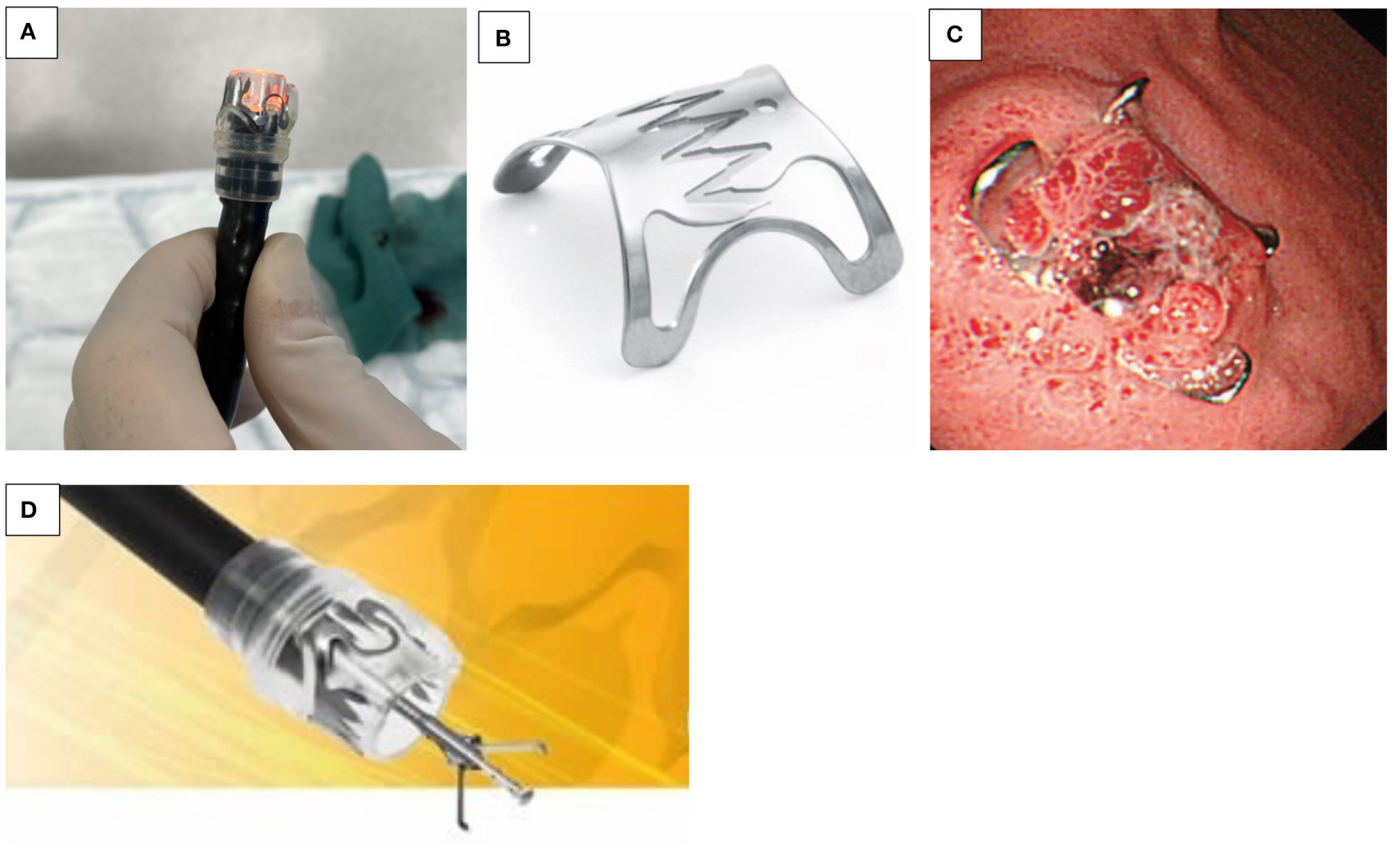
**(A–D)** Over the scope clip (OTSC^®^). **(A)** OTSC^®^ attached to the tip of the endoscope. **(B)** Grasping device of the OTSC^®^. **(C)** OTSC^®^ with its effective anchor mechanism. **(D)** Mucosal healing after OTSC^®^ application.

OTSC^®^ are now used in non-variceal bleeding, anastomotic dehiscence, perforation, and fistulae closure (e.g., IBD and post-gastrostomy removal). It is often proposed as the final option in endoscopic treatment before surgery (15). The OTSC^®^ system is composed of four components, including a grasper/clip device, a twin grasper, an “anchor” forceps, and a stiff tissue “brush.” The OTSC^®^ is attached on the tip of the endoscope, similar to variceal banding devices (Figure 2A). When compared to “through-the-scope” hemostatic clips, the primary benefit of its use in UGIB appears to be related to a combination of stronger tensile grasping strength of the jaws of the clip, a more effective anchor mechanism, and an improved size of tissue bite (Figures 2B, C). Additionally, the inter-clip space allows a continuous blood flow to the grasped tissue, preventing tissue necrosis during the tissue healing process (Figure 2D). In case of improper application, the novel alloy of the clip, Nitinol^®^, can be easily detached by the passage of electric current, with the aid of a specifically designed endoscopic cutting device. There are different sizes available, the smallest with a diameter of 8.5–9.8 mm, resulting in an intubation diameter of 14.6 mm, which is an issue in younger children. Kobara et al. recently reviewed a total of 1,517 OTSC^®^ cases with an average clinical success rate of 78%. In the case of anastomotic pathology, efficacy for prevention of rebleeding was 85%, and for fistulae, effective closure was 52% (15). A case series of seven pediatric patients has been reported (16).

##### Topical Hemostatic Endoscopic Approaches for GI Bleeding

Endoscopy for UGIB remains a challenging intervention, even for experienced endoscopists, as the incidence in children is relatively rare. A recent nationwide survey in the United Kingdom revealed that in the 16 tertiary Centers of Pediatric Gastroenterology, only 19% claimed that all their consultants were proficient in all endoscopic hemostatic techniques. Indeed 19% admitted that those interventions were beyond the technical capability of *any* of their staff. Only just over a half of the centers had an out-of-hours call service, of which 69% was covered by pediatric surgeons, who were also often unfamiliar with most of the techniques required (17). In this regard, a technique which is easily accessible even for less-experienced endoscopists is extremely valuable. Topical approaches lend themselves to lowering the threshold of endoscopic competency as they are so easy to apply—this may allow a wider and earlier hemostatic option.

**Figure 3:**
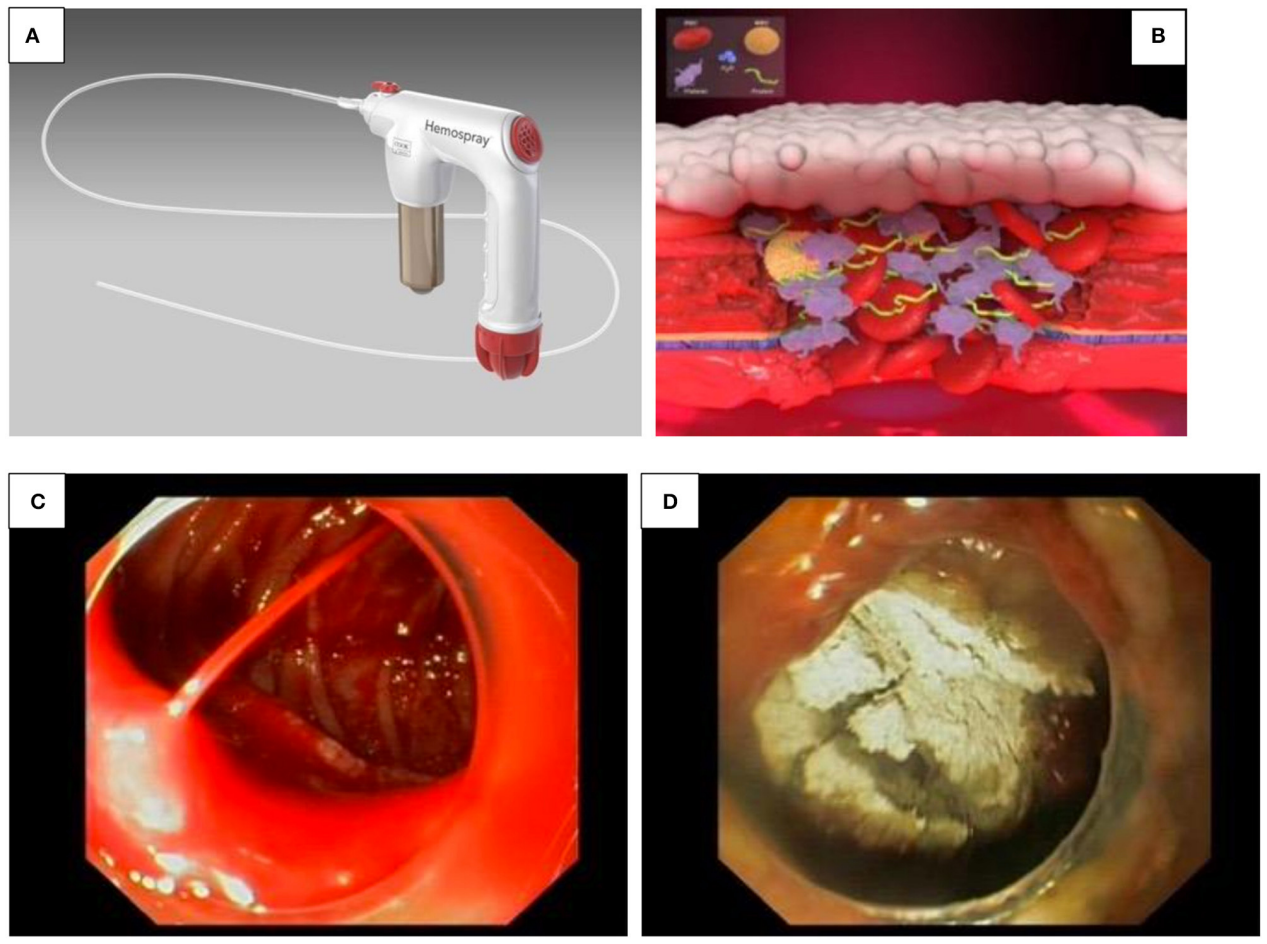
**(A–D)** Hemospray^®^. **(A)** Hemospray^®^ device. **(B)** Activation of the coagulation cascade by Hemospray^®^ results in immediate clotting. **(C,D)** Significant GI bleeding before **(A)** and after Hemospray^®^ procedure **(B)**.

A hemostatic spray (Hemospray^®^) is now licensed for non-variceal UGIB in the adult population in United States, Canada and Europe and has a CE mark (European approval) for its use in children (Figures 3A–D).

It is a highly absorptive, inert mineral powder, which functions as a mechanical tamponade by coagulation with the active GI bleed through the increase of clotting factors and the activation of coagulation cascade, resulting in immediate clotting (Figure 3B) (18). Besides its easy handling, one of its advantages is expeditious coverage of large surfaces (Figures 3C, D). However, the endoscope channel should be vigorously flushed with air prior catheter insertion as contact with any moisture might block the Hemospray^®^ catheter, making it unusable (17).

A recent meta-analysis of 11 prospective adult studies reported acute hemostasis of 93% in UGIB with 14.4% risk of rebleeding episodes. Hemostasis was nearly as successful in variceal bleeding with a rate of 92.7% and a rebleeding rate of only 3.1% (19). Another meta-analysis reviewed the efficacy of Hemospray^®^ in non-variceal UGIB and found a technical success in 97% of the cases treated with Hemospray^®^. In particular, in more recent studies (2011–2019), this compared favorably to 87% for other hemostatic measures (18). Thomson et al. prospectively enrolled 17 patients treated with Hemospray^®^ for UGIB and compared them to a second group, where conventional endo-hemostatic treatment had been applied. Both groups had achieved initial hemostasis in 100%, with 18% rebleeding in the Hemospray^®^ group, compared to 24% in the conventional group. The failure rate was also similar, with 6% for the former group vs. 7% for the latter (17).

Two new products, PuraStat (20) and EndoClot Plus (powder form), help reduce delayed bleeding following procedures such as GI endoscopic submucosal resection in the colon. EndoClot Plus (powder form) has also been used for treatment of bleeding ulcers (21).

### Foreign Body (FB) Ingestion

The management of FB ingestion can be a challenging situation for the pediatric gastroenterologist who has to determine the indication and timing for endoscopy, based on sometimes imprecise history, symptoms, and radiology.

This is usually in the under 5-year-old age group. FBs can be categorized in subgroups, such as blunt (e.g., different types of coins and toys), pointed/sharp (safety pins, nails, toothpicks, and hairpins), toxic (button batteries, magnets, drug packets, and caustics), and food impaction. Timing of endoscopy should be based on the clinical status, type and size of the FB, and if possible, the time of ingestion, last oral intake, and the location of the FB in GI tract.

Expert panels from Italy, ESPGHAN and NASPGHAN, suggested categories, with emergent (<2 h), urgent (<24 h), and elective (>24 h) (22–24). Depending on the FB and the age and size of the child, devices such as retrieval Roth nets, forceps (rat-tooth and alligator) polypectomy snares, tripod forceps, latex cones, and overtubes are used. To date, there are no pediatric studies comparing different retrieval devices (22). If the patient exhibits any signs of respiratory compromise, crepitus, neck swelling, or perforation, surgical consultation is mandatory (22–24).

#### Blunt Objects

Coins have been reported to be the most ingested object over a 10-year period in the United States, with over 250,000 ingestions with 20 deaths reported in younger children (<4 years old) likely related to airway blockage with a small coin (23). Depending on the size of the coin and the size of the patient, 30–60% may spontaneously pass through the esophagus into the stomach. Prior to endoscopy, biplane radiographs should be performed with careful inspection of the edges of the coin, to exclude a double-halo sign, which is suggestive of button battery (BB) ingestion, requiring immediate removal. Coins stuck in the esophagus should be removed within 24 h, in order to prevent esophageal injury or erosions into neighboring structures. However, if the child is unable to manage secretions or develops respiratory distress, then emergent retrieval is indicated (22–24). The coin or FB can be grasped with alligator-jaw forceps or rat-tooth forceps and retrieved back into the mouth; sometimes, however, it might be easier to gently push the FB into the stomach and grasp it there. If the coin/FB is located in the distal esophagus and endoscopy is not available, subcutaneous injection of glucagon might be used to relax the lower esophageal sphincter (LES) with spontaneous passage of the coin into the stomach. However, study results have been equivocal (24).

Once in the stomach, emergent endoscopy is generally not indicated for blunt objects except for those considered unlikely to pass the pylorus, e.g., between >2 and 3 cm for children younger than 1 year and between >3 and 5 cm for children older than 1 year (22–24).

#### Pointed/Sharp Objects

**Figure 4:**
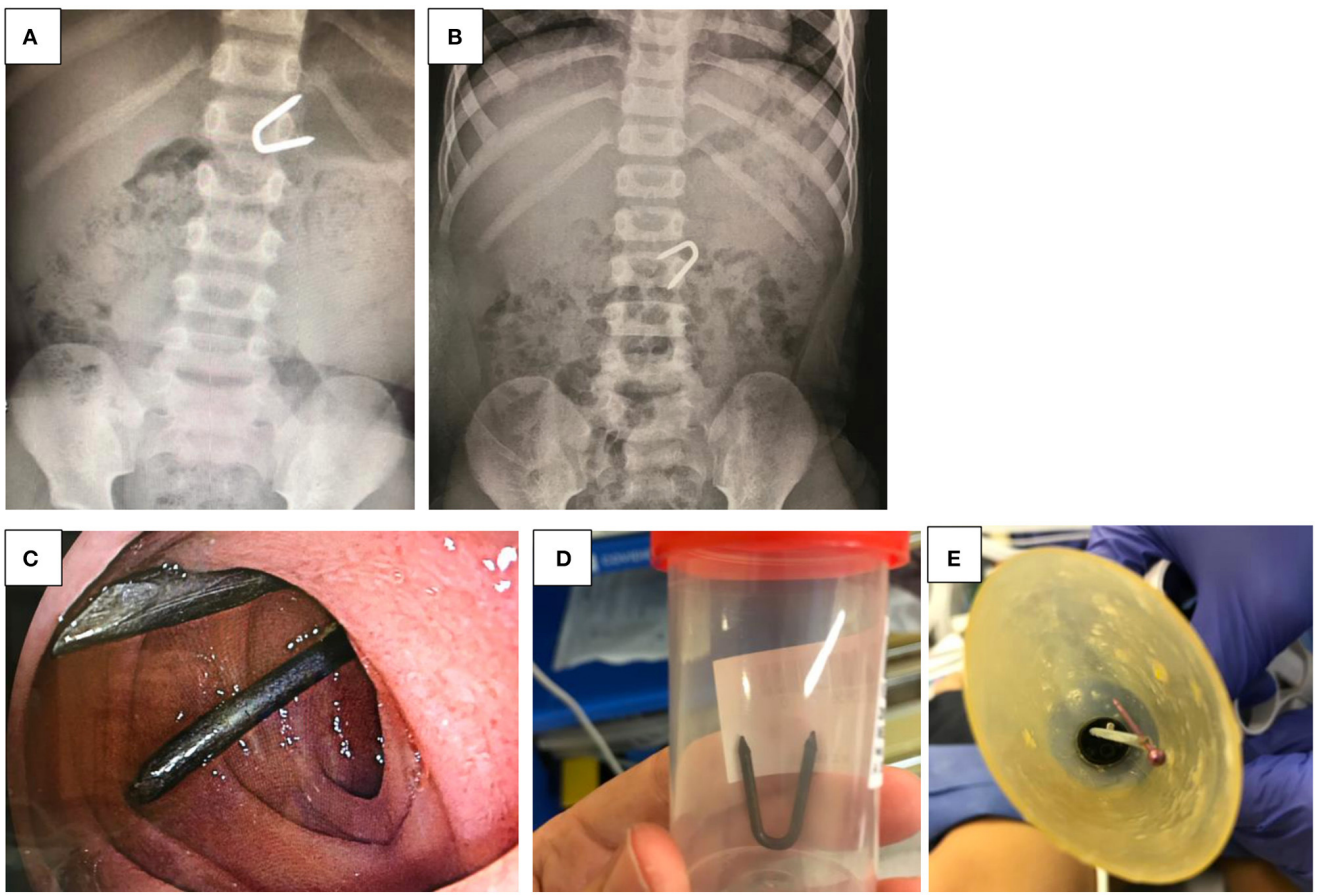
**(A–E)** Ingestion of a pointed foreign body. **(A,B)**. Abdominal x-ray of a pointed foreign body ingested by a 5-year old child. **(C)** Foreign body in the mid-duodenum. **(D)** FB after successful extraction with a retrieval forceps. **(E)** Foreign body (pin to fasten clothing) which has been grasped *via* a polypectomy snare and withdrawn into a protector hood.

The incidence of pointed or sharp FB ingestion has been reported to be between 11 and 13% in European and Asian centers (Figures 4A–E) (23). If the FB is located in the upper/mid esophagus, symptomatic ingestion tends to present with pain and dysphagia; however, up to half of the children can remain asymptomatic for weeks. Ingestion of toothpicks and bones are associated with a higher risk of perforation and are the most common FB requiring surgical removal (22). The main reported complications are perforation, migration into neighboring organs (liver, heart, lung, and bladder), abscess, and peritonitis, with the most common site of perforation being the ileocecal region (23). Prior to endoscopy, radiographic evaluation is crucial as it has a positive predictive value of 100% for metallic objects, but only 43% in glass and 26% in fish bones (Figures 4A, B) (23). If located in the esophagus, retrieval forceps, Roth nets, or polypectomy snares are useful retrieval accessories. However, if the sharp tip of the object is facing upwards, it might be safer to gently push the object into the stomach and retrieve it with the sharp part pointing downwards. Beyond the esophagus, an FB protector hood is a useful tool. It is attached to the tip of the endoscope and can be turned inside out by rubbing against the gastric mucosa (Figure 4E). The FB is then grasped with forceps or a polypectomy snare and then withdrawn into the protector hood and can then be safely removed. If the FB is located beyond the ligament of Treitz, either enteroscopic removal (if available) or surgery can be an option. If the patient is asymptomatic, observational monitoring might be considered but would need close follow-up with daily abdominal X-ray to assure continuous passage. It has been reported that the average transit time for FB in children is 3.6 days, whereas perforation occurred at a mean time after 10.4 days. Therefore, in case of non-progression after 3 days, surgery should be taken into consideration (22, 23).

#### Toxic Objects and Liquids

##### Magnets

While single magnets do not require endoscopic removal, the ingestion of two or more magnets presents an increased risk for the creation of an entero-enteric fistula between magnets located in adjacent bowel loops, leading to perforation, peritonitis, and necrosis, and these should therefore be removed. There has been an alarming report of The National Electronic Injury Surveillance System database in the United States, showing >16,000 estimated magnet ingestions in children between 2002 and 2011, which signifies an 8.5-fold increase of the incidence of magnet ingestion in children (23). It is therefore imperative to determine the number of magnets in the GI tract by obtaining at least two radiographic views of the chest or abdomen. Endoscopic retrieval nets are the best option for small round magnets. If conservative management is opted with an asymptomatic patient, daily abdominal X-rays should be performed, and in the case of non-progression, surgical intervention is the treatment of choice if enteroscopy is unavailable.

##### Button Batteries

BB ingestion accounts for between 7 and 25% of FBs ingested by children, most of them younger than 6 years, with a peak at 1 year of age. The incidence of BB ingestion has increased worldwide over recent years, and larger and more powerful batteries lead to a significant risk of severe morbidity and mortality, especially when impacted in the esophagus juxtaposed to large vessels—which has increased sevenfold in the last 20 years (25). When the BB comes in contact with the mucosa of the esophagus, the tissue serves as a conductor between the two battery poles, leading to H^+^ formation at the cathode, which results in the increase of pH with tissue liquefaction and necrosis. Damage might go beyond the esophageal wall, leading to fistulization into adjacent structures such as the trachea, aorta, and subclavian artery with sometimes life-threatening complications (22–25). Unfortunately, severe damage can already occur within 2 h after the first tissue contact. This is the reason why BB ingestion with impaction in the esophagus is *THE* emergency for a pediatric endoscopist *per se* and should not be delayed. Larger and newer BBs pose a greater risk for the creation of severe lesions, and even old batteries have the capacity to create a sufficient voltage to cause damage (25).

Biplane radiographs including the entire neck, chest, and the abdomen should be performed, and the image should be closely inspected with recognition of the “double-halo sign, ” as well as the step-off side, visible on the lateral film, indicating the cathode of the BB, which is the part causing most of the damage. A CT scan with contrast is usually indicated, especially in the case of delayed diagnosis with doubt about already-existing complications. This may need to be repeated the next day as aortic aneurysm may be delayed in its appearance.

Endoscopy should be performed, if possible, in the presence of a pediatric cardiothoracic surgeon, especially in the case of delayed diagnosis and a battery held up at the level of the aortic arch with esophageal ulceration at endoscopy. In the case of proximal localization, tandem work with the ENT team might be indicated. During endoscopy, meticulous inspection of the esophageal mucosa for localization, extension, and depth of the lesion is mandatory. If possible, the direction of the cathode (side without the “+” and without the imprint) should be determined, as it is generally the most affected site. The BB can be extracted either with a rat-tooth or alligator forceps or by using a retrieval net. If on X-ray the BB is already located in the stomach, endoscopic removal is only advised if it remains there after 7–14 days, as most of the BBs will pass the stomach during this period, rarely causing complications (25). If however the BB exceeds 20 mm, then spontaneous gastric passage is less likely, and therefore, these should be removed if still in place after >48 h (22).

In the case of severe lesions, repeat endoscopy should be performed at 24–48 h post-removal. Esophageal lesions can occur very quickly, but the development of complications may be delayed. If the anterior wall of the esophagus is affected, vascular and tracheal injuries are of great concern, whereas lesions in the posterior wall might lead to spondylodiscitis. Perforation generally appears within a 48 h time frame. Fistulization can even occur 4 weeks after removal, and other complications such as spondylodiscitis or laryngeal nerve damage can even take several weeks to months to occur (25). Horner's syndrome has also been reported as a complication of BB ingestion (26).

##### Caustic Ingestion

Fortunately, with the advent of child-unfriendly packaging of domestic products (such as detergents, softening, and dissolving agents), accidental ingestion of caustic products has significantly decreased in children (22). However, if agents are stored in non-original containers, ingestion of higher volume is possible, also called “accidental-deliberate ingestion, ” leading to potentially life-threatening conditions (24). The role of endoscopy is initially of pure diagnostic nature; following the Zargar classification (Table 2), esophageal lesions are classified as absent/mild till severe, and the subsequent treatment will be adapted following the Zargar grade, in order to prevent future complications, such as esophageal strictures.

**Table 2:** Zargar classification.

**Grade**	**Endoscopic finding**
0	Normal
I	Edema, hyperemia of the mucosa
IIa	Friability, hemorrhage, erosion blisters, exudates or whitish membranes, superficial ulcers
IIb	Grade IIa and deep discrete or circumferential ulcers
IIIa	Small scattered areas of necrosis, areas of brownish-black or gray discoloration
IIIb	Extensive necrosis

#### Other

##### Food Impaction

Compared to data in the adult population, where food bolus is the most common type of impaction, data in children are sparse (22–24). However, in most of the few studies existing, food bolus impaction in children tend to be secondary to underlying conditions, such as esophageal or reflux esophagitis, anastomotic strictures, achalasia, or other motility disorders.

If clearance is not spontaneous and the child cannot manage to secrete the impacted food, endoscopy should be performed in up to 24 h but may require urgent intervention if signs of near-complete obstruction occur (drooling and neck pain). Approaches like piecemeal or repetitive suction might be required. The latter can be performed by using the transparent cap of an EV banding device, which has been proven efficient in suctioning larger pieces of meat impaction (23). In some situations, a gentle push of the food bolus in the stomach might be an option but should only be performed if there is definite direct visualization of the esophageal lumen, as esophageal strictures or FB impaction might be present. Perforation can occur in up to 2% of the cases (24). After successful retrieval, esophageal biopsies are mandatory for the diagnosis of a potential underlying pathology. Hence, dilation may be delayed, contingent on the pathology leading to the impaction (22–24).

##### Gastric Bezoar

A bezoar is defined as a mass of accumulated substance found trapped in the GI tract, mostly in the stomach. The overall incidence of bezoars in children is unknown, and to date, only few studies exist, most of them case reports or case series. There are several types of bezoars with phytobezoars (composed of plant and vegetable components) being the commonest type (27). In comparison, trichobezoars are composed of hair, undigested fat, and mucus. The hair may come from the patient, other humans, animals, carpet fibers, or blankets. Hair fibers tend to get trapped in gastric folds, resisting peristalsis, as they are slippery.

**Figure 5:**
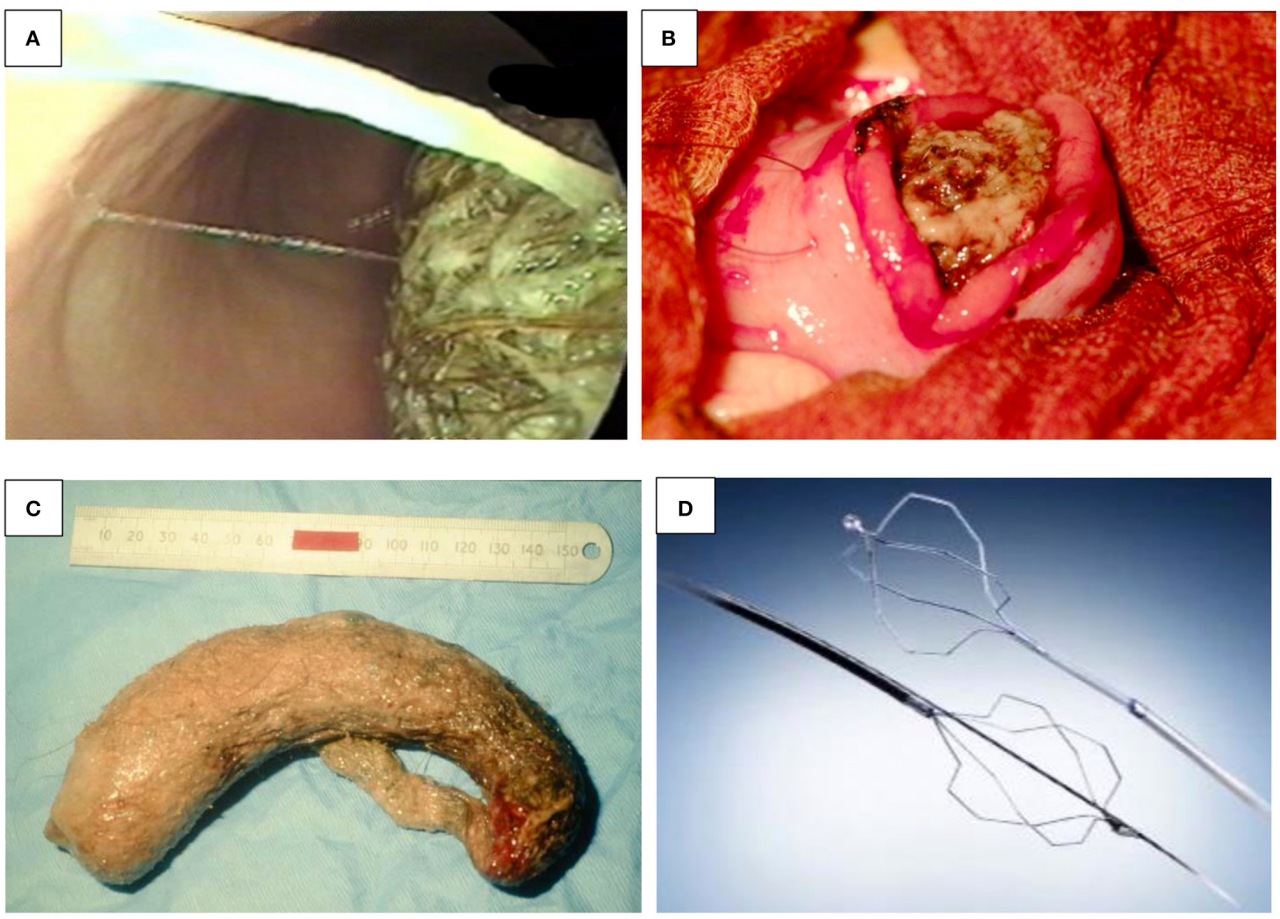
**(A–D)** Bezoars. **(A)** Bezoar seen at endoscopy. **(B)** Surgical removal of the bezoar from the same patient. Endoscopic removal wasn't possible. **(C)** A large bezoar removed. **(D)** Bezotriptor/Lithotriptor device.

One variant of trichobezoars is the “Rapunzel syndrome.” This is a trichobezoar extending from the stomach into the small intestine, sometimes even involving its entire length. The twisted hairs can become hard like a wire. There are reports in which these can cause compression of the mesenteric wall of the intestine, occluding the blood supply and resulting in pressure necrosis and perforations (Figures 5A–C) (28, 29).

The current management of gastric bezoars include dissolution (either by Coca-Cola beverages, cellulose, or papain), endoscopy, or surgery (laparoscopic and open) (27). With the help of an endoscope, the bezoar can be separated into smaller pieces using a polypectomy snare, biopsy forceps, directed water jets, injection of enzymes (papain and cellulose for phytobezoars), or mechanical lithotripsy (bazotome, a needle knife device, or bezotriptor, a lithotriptor), a device commonly used for the treatment of large bile duct stones (Figure 5D) (27, 30). Once the bezoar is broken into smaller pieces, these can then be either removed endoscopically or allowed to pass through the pylorus.

In a recent case series of 30 pediatric patients with gastric bezoars (one trichobezoar and the rest phytobezoars), the majority was removed by endoscopy, using a retrieving net (Roth net), generally requiring multiple passes (6–20). Four patients (13%) required surgery. Of note was a high prevalence of underlying GI disorders and dysautonomia in 20% of the children, suggesting that both are risk factors for gastric bezoars in children.

Even if the above-mentioned reports suggest a successful treatment of bezoars *via* endoscopy, endoscopic devices should be used cautiously. It is also important to know what is the constituent substance of the bezoar (31).

## Elective Therapeutic Upper GI Endoscopy

### Endoscopic Treatment of Pediatric Gastroesophageal Reflux Disease (GERD)

Gastroesophageal reflux (GER) is a common phenomenon especially in young infants and resolves in the vast majority in the first 2 years of life (32). However, if GER leads to troublesome symptoms that affect daily functioning and/or complications, it is defined as GERD (33).

If GER becomes GERD, management aims to achieve symptom relief while preventing complications. Patients who fail to achieve control with conservative methods may have persistent severe esophagitis or become dependent in the long term on anti-reflux treatments. In such cases, an anti-reflux procedure may be indicated (34). The principle of surgery in GERD is to reconstruct an anti-reflux barrier, although exactly how efficacy is achieved is not fully understood. Among several technical variants, the Nissen fundoplication is the treatment of choice to date. Its initial open approach has been replaced by laparoscopy since the early 1990s, but superior efficacy and safety have yet to be demonstrated in the pediatric population (35). In adult studies, complications are less commonly reported, success rate is good, and the laparoscopic procedure cosmesis is clearly superior (36, 37). Therefore, it could be argued therefore that there remains little or no place for open anti-reflux procedures in pediatrics.

A number of endoscopic techniques have been devised and used for treatment of pediatric GERD. These are described below.

#### Endoscopic Suturing Devices

Various endoscopic techniques have been developed in recent years, aiming to improve the function of the gastroesophageal junction (GEJ) to prevent GERD. We will briefly illustrate the different endo-suturing techniques, as most of them are now not used and most operators have translated their efforts on to Stretta^®^ (see below).

##### The EndoCinch Device

**Figure 6:**
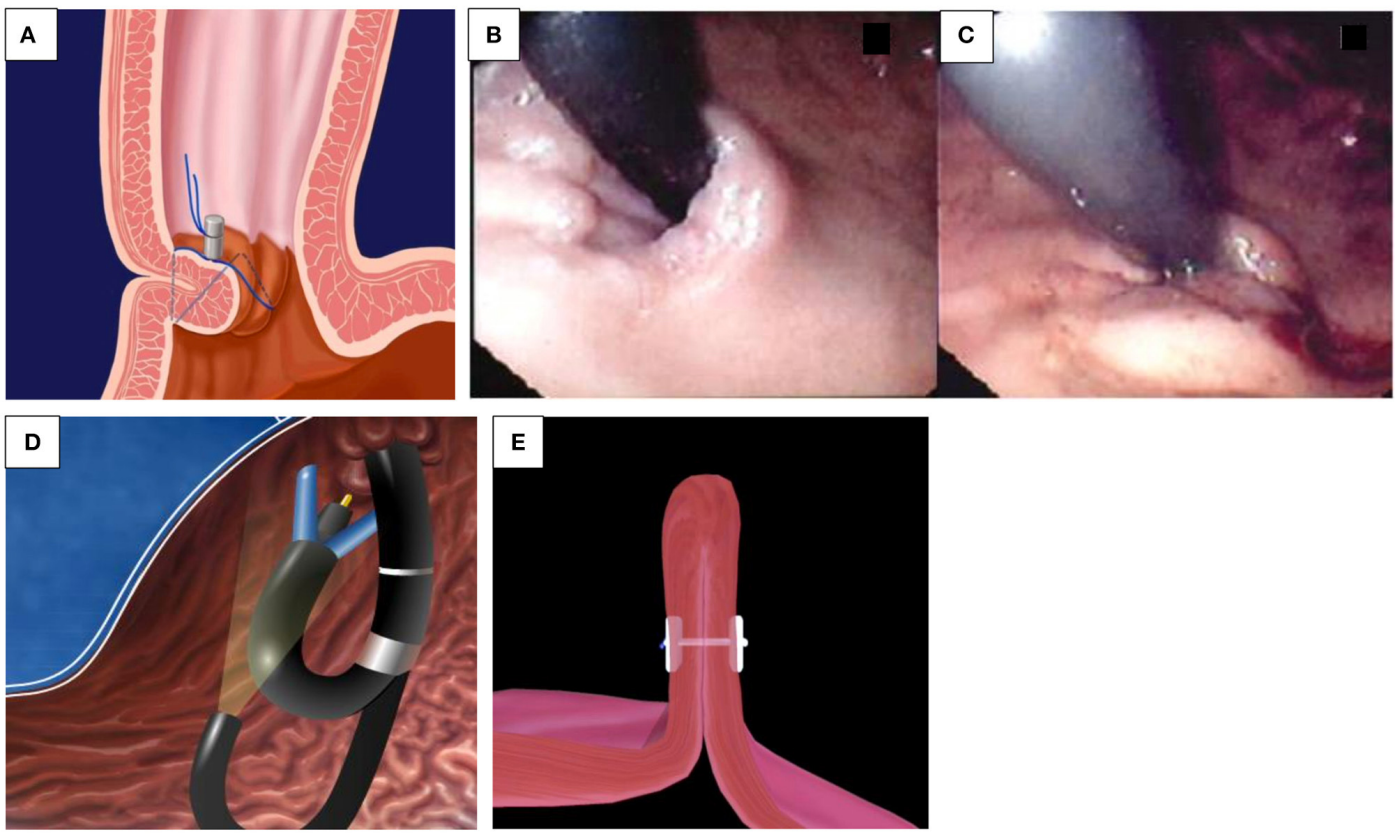
**(A–E)** Endocinch^®^ and full thickness Plicator^®^ (Ndo-Surgical). **(A)** Endoscopic gastroplication with a zig-zag stich when applied with an Endocinch^®^ sewing maching. **(B,C)** View (J maneuver) of a lax GO junction in a child with major reflux before **(A)** and after **(B)** application of stitch with the EndoCinch^®^. **(D)** Application of a full Thickness Plicator^®^ (Ndo-Surgical). **(E)** After application of the full Thickness Plicator^®^ (Ndo-Surgical).

EndoCinch is one of the historical endoscopic sewing systems, attached to the endoscope for the use of endoluminal gastroplication. Three pairs of stitches were placed below the GEJ, creating three internal plications of the stomach (38–40). According to the operator's preference, those plications may be applied in any manner, circumferentially or longitudinally (Figures 6A–C) (41). This is now historical.

##### Trans-oral Incisionless Fundoplication (TIF)

The TIF procedure using EsophyX mimics anti-reflux surgery in constructing an anterior partial fundoplication with tailored delivery of multiple fasteners during a single-device insertion (Figures 6D, E). The TIF procedure was designed to restore the anti-reflux competency of the GEJ through reducing small hiatal hernias, increasing LES resting pressure, narrowing the cardia, and recreating the acute angle of His.

In a meta-analysis, including seven trials with a total of 1,128 patients, TIF had the highest probability of increasing patient's health-related quality of life. However, it was not proven to be as efficient as the laparoscopic Nissen fundoplication in increasing LES, and based on the evaluation of benefits against risks, the authors did not recommend TIF as an alternative to PPI or fundoplication in the long term (42). This technology is now no longer available.

In summary, these trans-oral techniques are evolving and require further objective comparison with established laparoscopic fundoplication approaches in longitudinal prospective studies stratified for morbidity, in particular neurological compromise. Only then will the Stretta procedure be recognized as a viable alternative with its provisional advantages to date of being applicable to mainstream pediatric reflux management.

#### Delivery of Radiofrequency Energy (the Stretta^®^ System)

**Figure 7:**
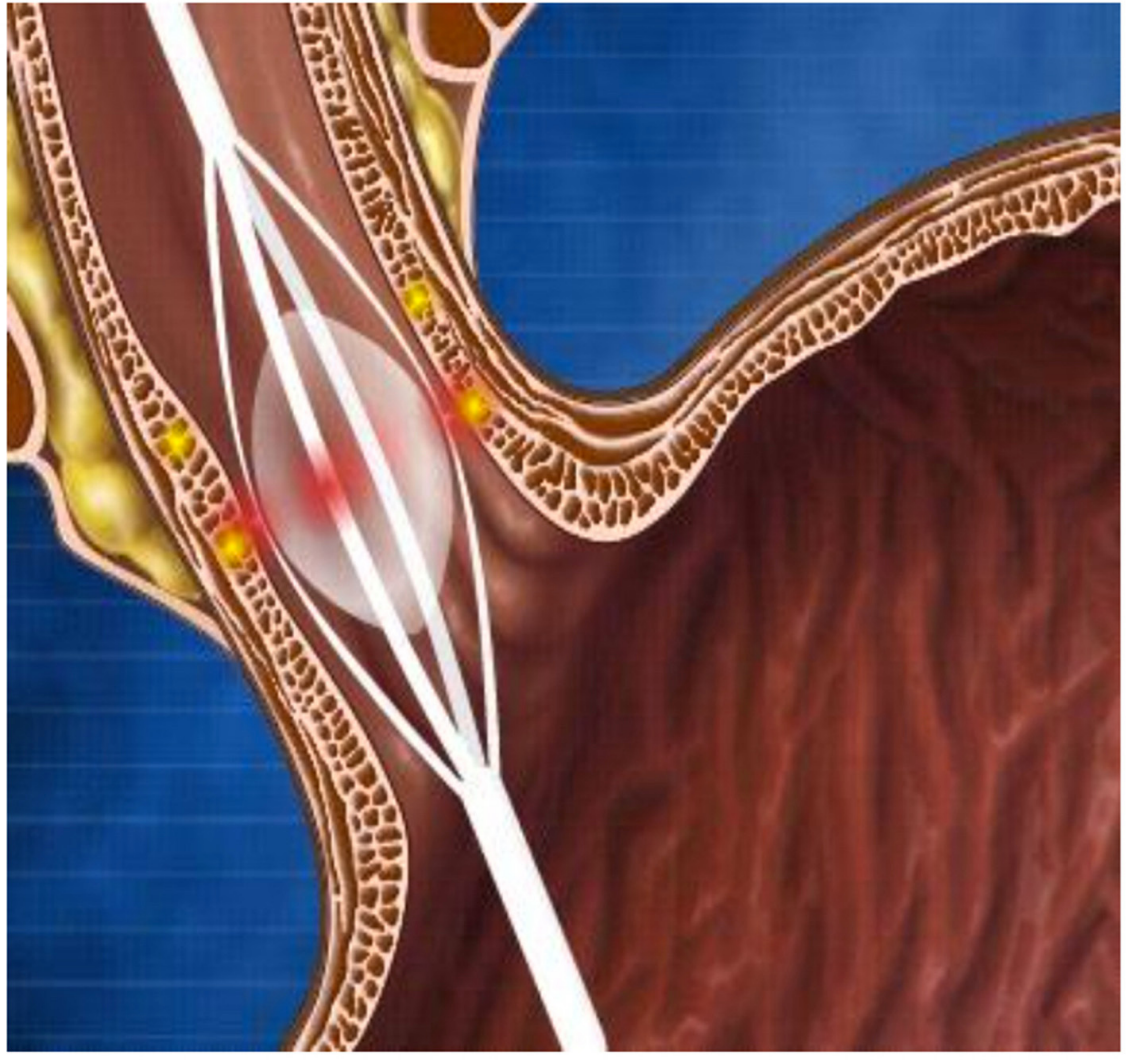
The Stretta^®^ procedure.

The Stretta^®^ procedure is a technique of tissue remodeling of the LES by delivering radiofrequency energy to the LES, muscle, and gastric cardia, hence improving the motility of the LES and its barrier function. The system has two parts: one a Stretta^®^ catheter and the other a Stretta^®^ control module. The Stretta^®^ catheter is a flexible, handheld, single-patient-use device that delivers radiofrequency energy generated by the control module (Figure 7). It is inserted over a flexible guidewire into the patient's mouth and advanced to the GEJ. A balloon is inflated, and needle electrodes are deployed into the tissue. Radiofrequency energy is delivered through the electrodes to create thermal lesions in the muscle of the LES and gastric cardia. As these lesions heal, the tissue contracts, resulting in a reduction of reflux episodes with improvement in symptoms. The Stretta^®^ control module delivers this radiofrequency, while at the same time providing feedback to the physician regarding treatment temperatures, tissue impedance values, elapsed time, catheter position measurement, and irrigation rate.

This treatment has been used in adults since 1999. Complications are rare and almost exclusively occurred with the first iteration of the device and not with the more recent device—but among those previously reported were ulcerative esophagitis with gastroparesis, esophageal perforation, and a case of aspiration following the procedure (43–45).

A recent meta-analysis including 28 studies involving 2,468 patients showed that Stretta^®^ significantly improved health-related quality of life and reduced heartburn. The mean follow-up was 25.4 (14–36.7) months, and reported adverse events were small in number, including small erosions in nine patients (0.36%), mucosal lacerations in seven (0.28%), gastroparesis in three (0.12%), and bleeding ulcer, mediastinal inflammation, pleural effusion, pneumonia each in one patient (0.04%) (46).

In pediatrics, the use of STRETTA^®^ was first reported in an uncontrolled study of a group of six teenagers (mean age 18.0 ± 3.4 years). These patients had a previous failed surgery (initial operation was 12 ± 4 years). Acute gastric distension was reported in one patient post-surgery and five of six were asymptomatic at 3 months' follow-up (43).

Liu reported the use of STRETTA^®^ in eight children (11–16 years) with a variable follow-up period of 5–15 months (47). It was reported that six of eight children improved (75%), and the cohort included three neurologically impaired children who also had concomitant percutaneous gastrostomy (PEG) placement. One of these groups had a post-procedure aspiration, which was successfully treated. Of the two failures, one remained dependent on PPI and the other had a successful Nissen fundoplication. Since this report in 2005, there have been no further publications of its use in children.

Although a recent meta-analysis shows the benefit of Stretta^®^ treatment for GERD (48), pediatric gastroenterologists may be guarded in using this form of treatment as clearly using thermal energy treatment in a 70-year-old is different from using it in a child who may have unknown consequences in the long term. An ongoing study in adolescents is occurring in our center.

### Advances in Endo-Dilatation for Treatment of Esophageal Stenosis and Strictures

Various etiologies can cause esophageal strictures and stenosis in children, with caustic, anastomotic, congenital, GERD, and eosinophilic esophagitis being the most common (22, 49–54). To date, there are various endoscopic treatment options, of which endoluminal balloon dilatation is probably the most useful and safe. Management focuses on long-term efficacy and safety, but the ideal timing of endoscopic dilatation remains a topic of debate. Recently, the initial recommendation of systematic subsequent dilatation every 3 weeks has been abandoned, and on-demand dilatation when symptoms occur is now the recommendation for benign strictures (22).

#### Esophageal Dilatation

The purpose of esophageal dilation is to alleviate symptoms and to permit free intake of enteral nutrition while reducing complications such as pulmonary aspiration. For dilation, two types of devices are available. One is the push bougie (Savary-Gilliard or Eder-Puestow) and the other the balloon dilator.

**Figure 8:**
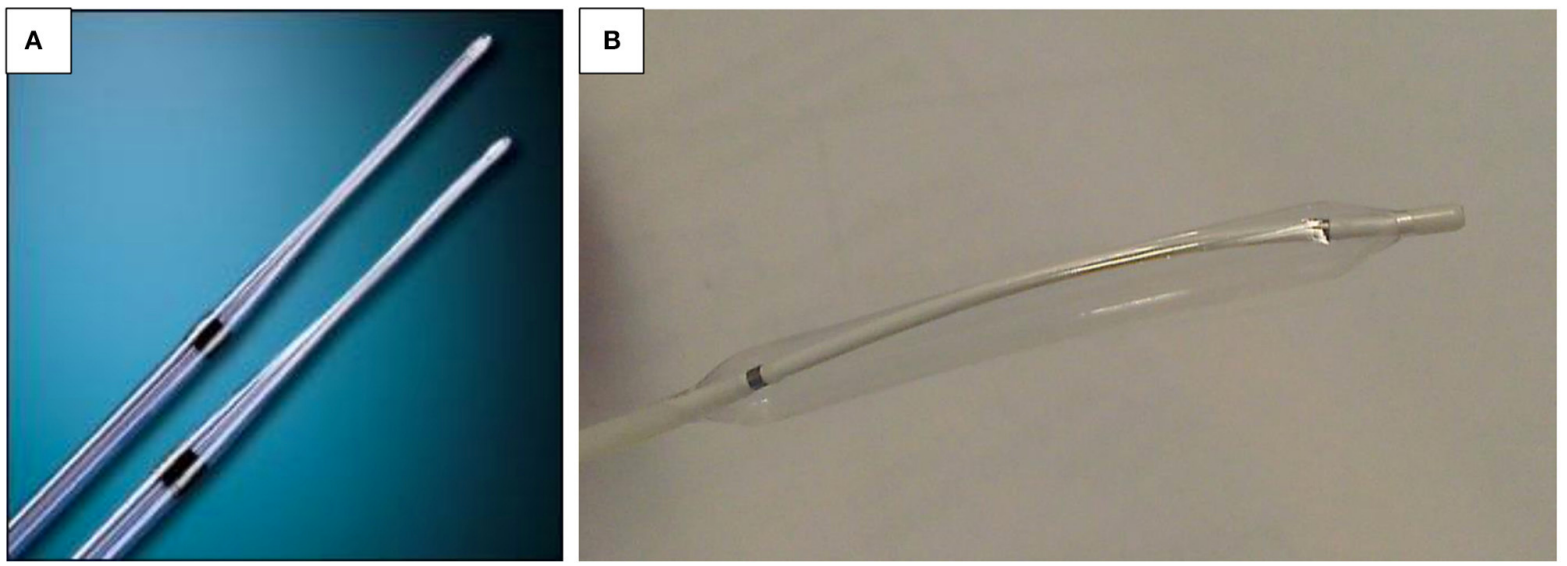
**(A,B)** Dilatation device. **(A)** Bougie dilator (Savary-Gilliard). **(B)** Balloon dilator.

Push dilators are made of rubber and may be weighted (tungsten/mercury filled) or wire guided (polyvinyl, metal, or Celestin type). The weighted dilators may be used blindly and vary in size from 7 to 20 mm (Figure 8A). It is generally agreed that unguided passage of weighted bougies should be used only in treatment of simple strictures and no more than two sizes for each dilatation session (55). Bougie-type dilators exert both radial and longitudinal forces due to the shearing effect, and balloon dilators exert a radial force. Due to this significant difference, it is recommended that radial balloon dilators are the tool of choice in children, with a lower rate of complications and equal efficacy, although prospective comparative studies are ongoing.

The balloon dilators may also be wire guided, or they may be passed through the endoscope. These vary from 4 to 20 mm (Figure 8B). It is suggested that a guidewire should be placed under direct vision. The authors, in common with most endo-therapeutic practitioners, prefer balloon dilation under direct vision with the balloon centered at the tightest point of the stricture.

The threshold for screening should be low, and fluoroscopy during the procedure is recommended in most cases and especially when using non-wire-guided dilators, during dilation of complex esophageal strictures, or in patients with a tortuous esophagus (22).

To reduce the risk of perforation, it has been suggested that no more than three dilators of progressively increasing diameter should be passed in a single session (56). The “rule of three” also suggests that no more than a three-fold increase in luminal diameter is attempted each time.

Esophageal perforation is a worrying complication of dilation therapy, with a global risk for perforation between 1.5 and 2.6%, according to different observations. The “rule of 3, ” to prevent perforation, has been adopted from the ASGE recommendations and implies that the dilation of a stricture should not be greater than three times the diameter of the stricture. However, Clark et al. have recently challenged this recommendation for children with stenosis of esophageal anastomosis: by reviewing charts from 284 children who underwent in total 1,384 balloon dilatations, they observed that dilatation of ≤ 5 mm did not unduly increase the risk of perforation, with a cumulative rate of perforation for dilatations ≤ 5 mm of 0.74%, whereas the risk increased to 4.85% in dilations ≥6 mm (49).

Readily available pediatric surgical support is vital while performing this procedure in children. Adult studies show that the risk of perforation is four times higher if the endoscopist has performed <500 therapeutic endoscopies (57).

Perforation should be suspected in any child developing continued chest pain, breathlessness, fever, or tachycardia. A chest X-ray is a useful first-line investigation. This is particularly true if the stricture is man-made, i.e., anastomotic, as perforation is more likely in such a situation.

#### Adjuvant Treatments With Dilatation

Dilatation of esophageal strictures creates a repetitive local mechanical trauma which may result in the stimulation of fibrogenesis and additional collagen disposure and therefore formation of fibrosis and scar tissue, resulting in stricture recurrence (51, 53). Several adjuncts to esophageal dilatation are nowadays in use to prevent stricture recurrence, which are detailed below.

##### Intralesional Steroid Injection

The intralesional injection of triamcinolone acetate has been studied in adults and in children without convincing results. However, Ngo et al. recently observed a significant increase in stricture diameter in 158 patients with anastomotic strictures post-esophageal atresia, with triamcinolone acetate and balloon dilatation compared to those treated with dilatation alone. However, benefit was limited to the first three dilatations (53). Therefore, intralesional injection of triamcinolone acetate might be an option for refractory esophageal stricture but should be limited to three procedures.

##### Use of Mitomycin C (MMC) Following Dilation

Recurrent stricturing due to any cause should suggest the use of an anti-fibrotic topical treatment post-dilation. Circumferential or deep caustic burns have a poor outcome, with an increased risk of perforation and/or stricture formation, even with early steroid treatment.

Thomson et al. reported the first use of MMC in a child with caustic stricture necessitating recurrent dilations (58). An 18-month-old girl at that time developed two strictures after accidental ingestion of caustic soda and was treated with dilation many times before topical application post-dilation of MMC, preventing the need for further dilation. At 20 years' follow-up, she is asymptomatic.

Since the publication of this first report, MMC has been used worldwide in different pathologies, e.g., caustic, post-surgical stenosis, and epidermolysis bullosa strictures (59). A French multicenter study showed a 67% success rate in their 39 patients with a significant decrease in number of dilatations prior (102) and post MMC application (17) (51). Wishahy et al. observed a significant improvement in dysphagia score in their 17 children treated with MMC (54). In general, patients received an MMC dose between 0.1 and 1 mg/ml. It is not known if the early use of MMC is more beneficial.

#### Electrocautery Incisional Therapy (EIT)

Another option for the management of refractory esophageal strictures is endoscopic EIT, which has been reported in adults and has recently been successfully employed in children by Manfredi et al. (52). A total of 133 EIT have been performed for 58 anastomotic strictures in 57 pediatric patients, subdivided into refractory (36) vs. non-refractory strictures (22). Treatment success, defined as no requirement for stricture resection, appropriate diameter for age, and less than seven dilatations in 24 months, was achieved in 61% in the refractory group and in 100% in the non-refractory group (52). Performed by an experienced endoscopist, EIT might be an interesting option, especially in asymmetric strictures, where balloon dilatation with exertion of equal force in all direction might tear less dense tissues easily. Manfredi et al. used a needle knife to incise strictures at their most obviously dense part, followed by a second incision and balloon dilatation to cause tearing at the incision site, hence fortifying the incision and dividing the fibrotic tissue. However, perforation occurred in 2.3% without the need for surgical intervention but was higher than that in most of the cohorts with simple balloon dilatation. Therefore, performance only by an experienced endoscopist and in conjunction with a surgeon is recommended.

#### Fully Covered, Self-Expandable Metal Stent (FCSEMS)

**Figure 9:**
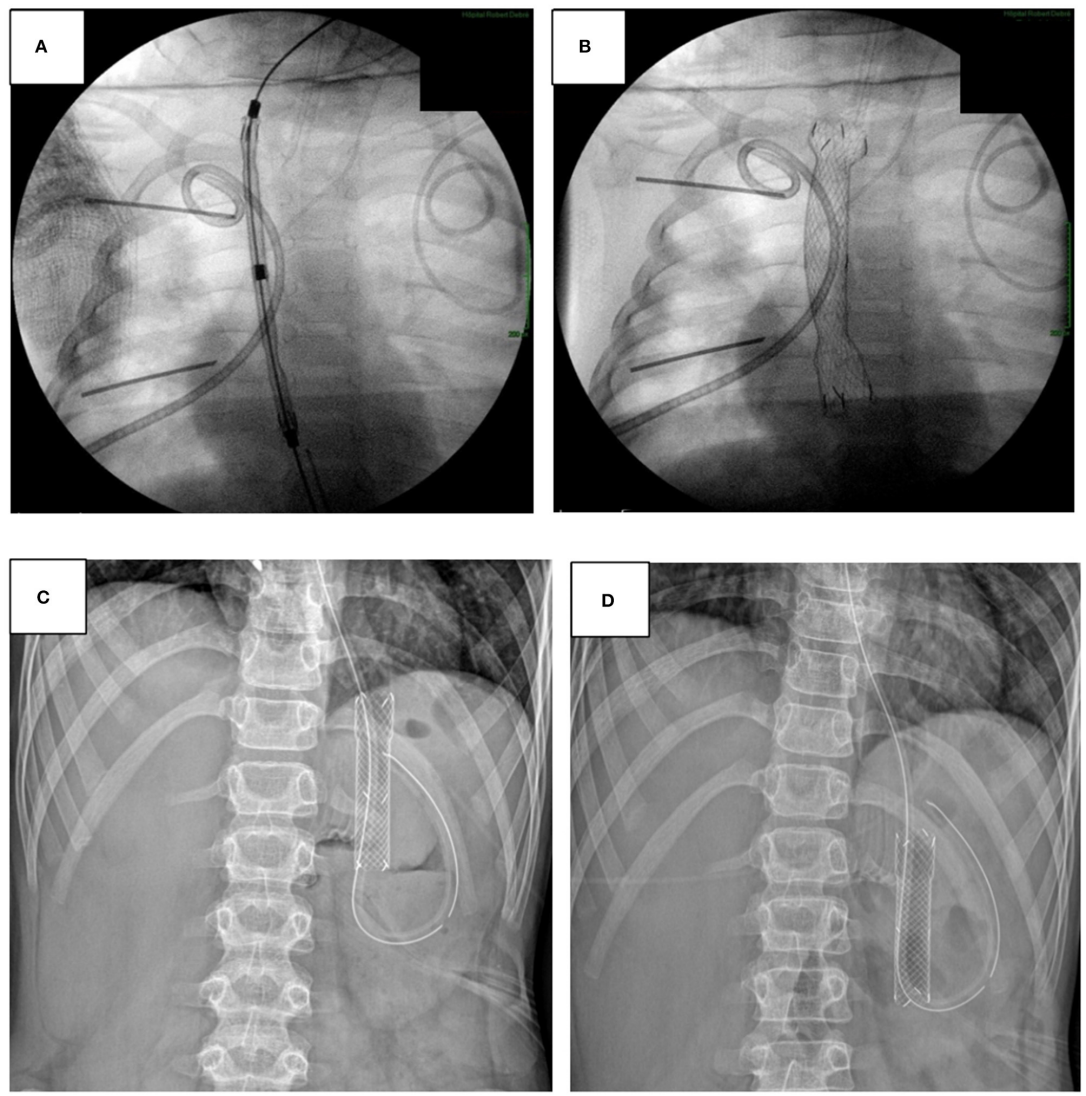
**(A–D)** Fully covered, self-expandable metal stent (FCSEMS) with the courtesy of Prof. Jérôme Viala, Robert-Debré University Hospital, Paris, France. **(A)** Insertion of a FCSEMS the esophagus *via* a guide-wire. **(B)** FCSEMS after expansion. **(C)** FCSEMS placement in a 12 year old child after Toupet perforation. **(D)** Displacement of the stent in the stomach, requiring insertion of a longer stent with afterwards satisfying hermeticism and closure of the perforation.

FCSEMSs have been used for refractory esophageal stenosis in children and in adults (Figures 9A–D). In three pediatric studies, including in total 25 patients, complete clinical response (no recurrence of dysphagia or need for subsequent dilatations) after stent removal was achieved in 50–85%. However, the most frequent adverse event was stent migration, which occurred in up to 29% (22).

FCSEMS also represents an attractive therapeutic option for the management of anastomotic leaks after esophageal or gastric surgery (Figures 9C, D). Sometimes, especially after multiple complex surgical procedures, conservative treatment (using broad-spectrum antibiotics, drainage, and parenteral nutrition) might be indicated, and FCSEMS has emerged as a promising minimally invasive option in adults to promote leak closure. In a recent case series of 10 children with post-surgical anastomotic leaks, perforation closed in 9 of 10 patients but 4 of 9 developed subsequent stenosis after stent removal (60).

### Endoscopic Treatment of Barrett's Esophagus

Barrett's esophagus is a complication secondary to chronic acid exposure/reflux esophagitis resulting in columnar metaplasia of cells in the distal esophagus extending ≥1 cm proximal to the GEJ. Barrett's esophagus is a worrying condition as it is considered to be a major predisposing factor for development of adenocarcinoma conferring a 0.5% to 7% lifetime risk of developing malignancy, or approximately 0.66% per year in the adult population after development of dysplasia (61–63). Compared to prevalence in adults, that in children and adolescents is very low, ranging from 0.055 to 0.13% (63). It is an uncommon condition in children, but there is evidence of genetic predisposition in one pediatric study (64).

Identification of the GEJ is important, and biopsies are taken following the Seattle protocol (62). Over the years, several techniques have been developed, through which successful ablation is proposed: use of Nd-YAG laser (65, 66), KTP (potassium titanyl phosphate) laser (67–69), multipolar electrocoagulation (70, 71), APC (72–74), and photodynamic therapy (67, 75, 76). These techniques have been little used in pediatric practice except anecdotally, and the details of each are beyond the scope of this chapter.

### Peroral Endoscopic Myotomy (POEM)

Achalasia is a rare progressive motility disorder, characterized by esophageal aperistalsis and impaired LES relaxation, leading to increased dysphagia of solids and liquids and regurgitation of indigested contents (77–82). Its presentation is particular in adult life, and diagnosis in childhood is quite rare. Achalasia is not curable, and treatments focus on the reduction of LES pressure. Current management includes laparoscopic Heller myotomy (LHM), POEM, pneumatic dilatation, and the injection of botulinum toxin. Since its first description in 2010 by Inoue et al., POEM has become an effective and safe procedure worldwide with the advantage of significant lower operation time and a shorter length of stay, and hence, it has replaced LHM as first-line treatment in adults (77, 82).

**Figure 10:**
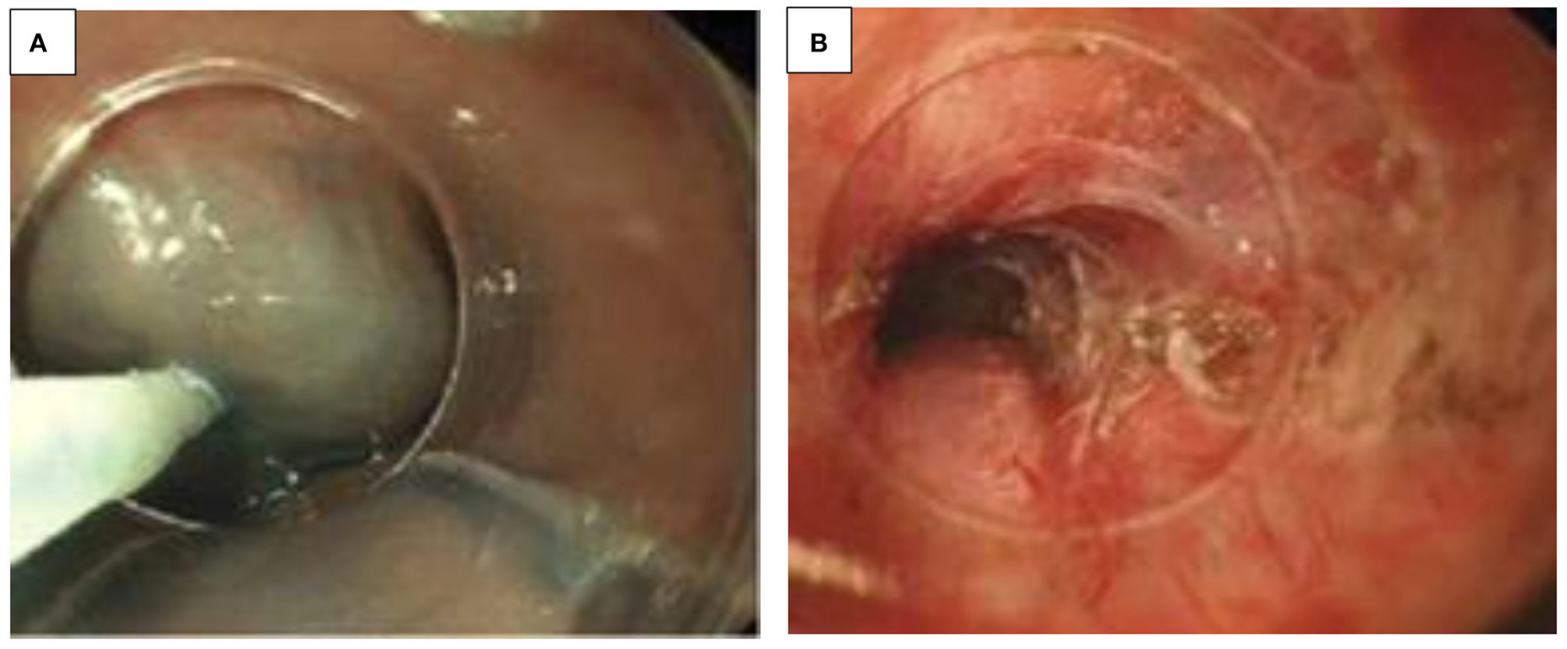
**(A,B)** Peroral Endoscopic myotomy (POEM). **(A)** POEM procedure: Incision of the submucosal bleb to create a submucosal tunnel. **(B)** Myotomie during POEM procedure.

After endoscopic identification of the GEJ, a submucosal bleb is created by the injection of saline-indigo or methylene blue solution in the mid-esophagus. Then a 1.5–2 cm longitudinal incision is made, using either a dual, triangular-tip, or hook knife (Figure 10A). A submucosal tunnel is then extended to the gastric cardia, using minimal electrocautery, methylene injection, or blunt dissection.

Myotomy is performed starting at 2–3 cm distal to the mid-esophageal incision with either full thickness or circular dissection onto the proximal cardia approximately 2 cm below the GEJ (Figure 10B). An endoscopic clip is placed to close the entry site at the end of the procedure.

Clinical success rates in adults vary between 82 and 100% with particularly good results in patients with prior failed therapy: a recent meta-analysis indicates a 98% success rate in patients who had failed LHM (77). POEM has now been reported in children. A recent multicenter study, including 117 pediatric patients, showed clinical success in 90.6%, with only seven adverse events (6%), including mucosotomies, subcutaneous emphysema, and one esophagopleural fistula (79). A recent meta-analysis, including 12 studies with 146 pediatric patients, revealed a significant reduction of clinical symptoms and LES pressure, with at least 93% of the patients experiencing improvement post-POEM (78).

Owing to well-established training programs for this highly technical procedure, the perioperative complication rate is very low in the adult population. However, GER secondary to POEM is observed, ranging between 15 and 19% in the pediatric and adult populations, respectively (79).

### Endoscopic Pyloromyotomy for Congenital Pyloric Stenosis

Ramstedt's pyloromyotomy (open and laparoscopic) has been the gold-standard operation for treatment of congenital hypertrophic pyloric stenosis (CHPS) for more than 80 years. Ibarguen-Secchia from Texas has reported the use of endoscopic pyloromyotomy in a series of 10 children (83). This was performed with a view to achieving a quicker operation and postoperative recovery time. Of the 10 children, nine had the procedure as a day case and one needed electrolyte correction before being treated the next day. All children were fed after only 11 h following the procedure compared to the median time of 38 h for laparoscopic pyloromyotomy and 64 h for an open abdominal procedure. Vomiting continued to a lesser degree in two but eventually resolved in all over 6–18 months' follow-up. Zhang et al. treated nine infants with CHPS, using an endoscopic electrosurgical needle knife. All patients started feeding 2–10 h after the intervention. There was a resolution of vomiting after 1 week in eight of nine patients. One child required a second endoscopic pyloromyotomy related to recurrent vomiting (84).

Despite these promising case series, indicating that endoscopic pyloromyotomy is a safe, effective, and minimally invasive procedure, there are no further recent case series about endoscopic pyloromyotomy, which is probably related to a very safe and effective surgical procedure. To date, laparoscopic pyloromyotomy remains the treatment of choice in most pediatric centers, but pre-pyloric congenital webs and peptic/caustic pyloric stenosis have been treated endoscopically in children.

### Percutaneous Endoscopic Feeding Tubes: Gastrojejunostomy

Percutaneously placed feeding tubes can be used in various techniques, ranging from PEG, single-stage PEG (SSPEG), percutaneous gastrojejunal (PEGJ), and direct laparoscopic-assisted percutaneous jejunal (LAPEJ) tubes. Standard and SSPEG insertion is not covered in this paper as it is such a widespread technique and is covered in detail in textbooks (8).

In children with severe GERD and/or gastroparesis, post-pyloric feeding might be indicated. With the PEG tube, it is also now possible to place a PEGJ tube. A thinner jejunostomy tube is placed through the PEG tube lumen. The jejunostomy tube then traverses the pylorus and extends down beyond the ligament of Treitz. Gastrojejunal button devices are also available in two different lengths and sizes, for children under and over 10 kg, and can be placed as an initial procedure. Unfortunately, gastrojejunal tubes are fraught with problems and tend to get blocked or displaced easily, requiring recurrent radiological and/or endoscopic replacement and necessitating either radiological exposure or general anesthesia. However, complication rates vary widely, and complications such as displacement or obstruction depend not only on the endoscopist but also on the training and experience of the care team who handles the enteral nutrition devices. Direct surgical procedures (in general modified Roux-en-Y jejunostomies) have the disadvantage of being more invasive and related to a higher rate of complications (85). In general, working hand in hand with the pediatric surgeon is essential, in particular in children with reflux disease and/or requiring enteral feeding access, as these children usually have complex comorbidities which could potentially require surgical assistance.

**Figure 11:**
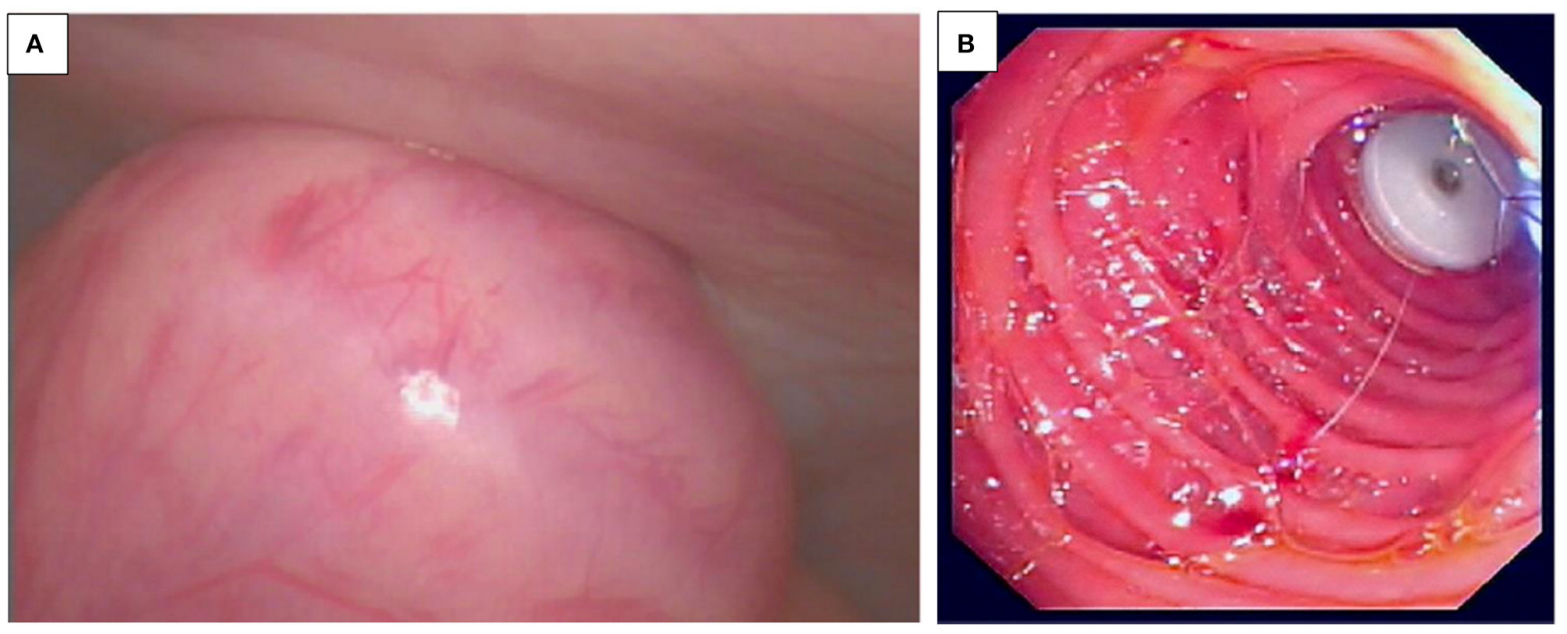
**(A,B)** Laparoscopic-assisted percutaneous endoscopic jejunostomy (LAPEJ). **(A)** Laparoscopic view of the proximal jejunum pulled to the abdominal wall by PEG tube insertion. **(B)** Endoscopic view of the Corflo in the jejunum.

A minimally invasive technique combining endoscopic and laparoscopic approaches has recently been reported, which allows the direct insertion of a jejunostomy using simultaneous endoscopic and laparoscopic visualization to maximize safety and potentially improve outcome (85). The LAPEJ involves the following steps: insertion of the endoscope by the endoscopist into the proximal jejunum while the surgeon uses a laparoscopic camera and one or two additional instruments to identify the duodenojejunal flexure and clamp the distal jejunum to prevent excessive insufflation of small bowel obscuring the laparoscopic view (Figure 11A). A trocar is then inserted to introduce a wire across the abdominal wall into the jejunum, which is visualized, grasped, and retrieved by the endoscopist followed by a standard “pull” technique to bring a PEG tube out through the jejunum, across the peritoneal cavity, and out of the skin. Placement is confirmed endoscopically and laparoscopically (Figure 11B). In a case series of 16 patients, the LAPEJ procedure has been proven a safe, effective, and minimally invasive technique to achieve medium- to long-term direct jejunal access for feeding and could be completed in a short operative time (85).

### Endoscopic Mucosal Resection (EMR)

EMR was originally described by Deyhle et al. and has been developed by Japanese endoscopists for the resection of sessile and flat lesions of the upper GI tract in adults and children (8, 85, 86). EMR is now an established standard procedure for sessile polyp removal in adults with the advantage of avoidance of thermal damage and reduced procedure times (87, 88). It permits the resection of flat and sessile lesions by longitudinal section through the submucosal layer (89). The European Society of Gastrointestinal Endoscopy (ESGE) recommends EMR with a cold snare in diminutive polyps (≤ 5 mm) and sessile polyps up to 9 mm (88). In a retrospective analysis, Zhan et al. compared two case series of patients treated with either high-frequency electrocoagulation (HFEC) or EMR. Operation and intraoperative and postoperative bleeding were similar in both groups, without any perforation. Only hospital stay was longer in the EMR group compared to the HFEC group (90). EMR facilitates complete histological analysis of the resected lesion and makes it possible to determine precisely the completeness of excision in both the horizontal and vertical resection planes. This makes it advantageous compared to primary tissue ablative techniques such as APC (91) and electrocoagulation (92). Numerous EMR techniques have now been described using transparent caps fitted to the proximal aspect of the endoscope and using an insulation-tipped cutting knife (86).

### Botulinum Toxin Injection

#### Esophagus

Botulinum has been used for treatment of achalasia of the esophagus, but the symptom relief when achieved is only short-lived. Functional esophagogastric junction obstruction with intact peristalsis (in the absence of achalasia) has been described in adults (93).

#### Pylorus

Botox has been used in the pylorus to help delayed gastric emptying. In a 32-patient randomized-controlled trial (RCT) in Philadelphia, intra-pyloric injection of botulinum toxin improved gastric emptying in adult patients with gastroparesis, although this benefit was not superior to placebo at 1 month (94). A systematic review on intra-pyloric botulinum toxin injection for gastroparesis confirms the findings of the RCT (95). The authors have successfully used botulinum toxin injection in children in the esophagus and the pylorus.

#### Sphincter of Oddi

Following an initial report of successful use of botulinum toxin in the bile duct of a canine model to decrease biliary pressures (96), it has been used for relaxation of the sphincter of Oddi in selected patients with acalculous biliary pain (97). The pain relief was followed by sphincterotomy in the responders and cholecystectomy in the non-responders.

### Pancreatic Pseudocyst Drainage

Pancreatic pseudocysts are secondary to pancreatic damage and may be multi-etiological: traumatic; post-pancreatitis of idiopathic origin; following chemotherapy; or any other cause of acute pancreatitis. They should be differentiated from malignant cysts, but this is unusual in childhood and is a distinction necessary predominantly in adult practice.

Presentation may be with a persistently raised amylase, with chronic pain, as an abdominal mass, or with consistent nausea/vomiting. Treatment to date has been either conservative or surgical, with the use of anti-secretory agents such as octreotide or its longer-acting analogs (e.g., lanreotide) or *via* ERCP.

**Figure 12:**
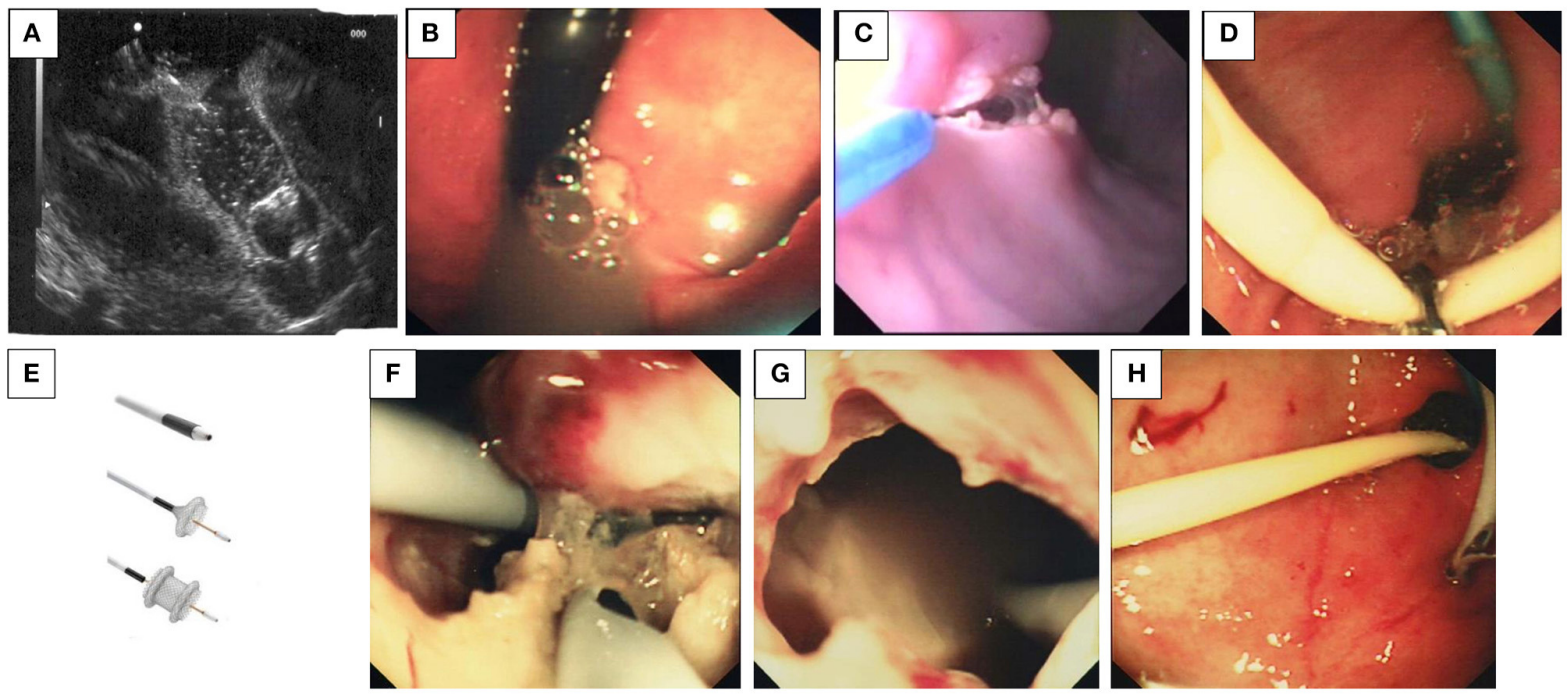
**(A–H)** Drainage of pancreatic pseudocysts. **(A)** Trans-gastric linear endo-ultrasound needle puncture of a pancreatic pseudocyst. The linear needle can be seen as a straight white line in the upper part of the picture. **(B)** The indentation into the gastric wall can be seen easily identifying the position of the pseudocyst. **(C)** Creation of a cauterized entry from the stomach into the cyst by using and endoknife and sphincterotome: After endo-ultrasound has identified the cyst and a site which is free from gastric vessels, an endoknife followed by a sphincterotome (tapertome is best) is used to create a cauterized entry point from the stomach in to the cyst. Adrenaline can be injected prior to the incision to further diminish the possibility of hemorrhage during incision. **(D)** Grasping forceps are used to manipulate the stents [pig-tailed (blue) or straight (white)] through the gastro-cystostomy that was created. **(E)** Self-expanding metal stents. **(F)** The stents are endoscopically observed in the pseudocyst, and membranes between loculations can be punctured as necessary. **(G)** The endoscope is withdrawn from the pseudocyst. **(H)** The endoscope is withdrawn from the stomach and the gastro-cystostomy is left in place.

More recently, trans-gastric cystostomies have been performed by endoscopy (98). These are either guided by endo-ultrasound (EUS), which may be a safer option by avoiding gastric vessels (Figure 12A), or blind with prior epinephrine injection into the bulge in the gastric wall from the luminal surface and then incision into the injected area. Indeed, EUS has become the accepted guidance approach for drainage of pancreatic fluid collections in the past decade. EUS has been shown to be safe and effective, and it has been the first-line therapy for uncomplicated pseudocysts. Where walled-off pancreatic necrosis was originally thought to be a contraindication for endoscopic treatment, multiple case series have now shown that these fluid collections also can be treated endoscopically with low morbidity and mortality (99). Usually, the cyst can be indirectly identified abutting the lesser or greater curvature and is quite obvious as a mass effect into the gastric lumen (Figure 12B).

The initial incision may be made with an endo-knife (Figure 12C), and once this is made, a sphincterotome may be inserted and employed to safely expand this incision. However, this has the disadvantage of then obscuring the endoscopy view with an outpouring of a great deal of fluid. A better approach is to use a cystotome which requires a 3.2 mm working channel in the endoscope but which prevents loss of access to the cyst—this is because the endo-knife is within the cystotome and the incision and then introduction of a guidewire can be seamless—the stents can then be passed down the 3.2 mm working channel and into the cyst with the proximal portion in the stomach. Subsequently, either straight ERCP plastic stents or pig-tailed stents can be inserted into the pseudocyst and left *in situ* (Figure 12D). Recently, the temporary placement of self-expanding metal stents has been reported (AXIOS, Figure 12E) (100, 101). Fluid will then follow the path of least resistance, and the presumed communication with the pancreatic duct will close, preventing further accumulation of pancreatic fluid in the cyst. An endoscope may be inserted into the cyst, but this is not strictly necessary (Figures 12F, G). It is hoped that the gastric wall and the cyst will become adhesive and fibrotic, creating a channel such that the stents become unnecessary as when the cyst naturally deflates, the stents are extruded and the fistula closes (Figure 12H). This is the normal course of events. Patient symptom relief is acute and usually long-lasting. Complications are not common as long as gastric vessels are avoided initially. A combined approach involving drainage through the papilla and transmural endoscopic drainage can be useful in the larger and more loculated cysts. The efficacy and safety of this procedure in the pediatric population have been described by utilizing ultrasound-guided drainage (102).

### Endoscopic Treatment of Obesity

The endoscopic treatments for obesity include space-occupying devices (balloons), endoscopic techniques that reduce gastric capacity (suturing methods for plication and partition), endoscopic treatments modifying gastric motor function (injections and implants), and use of malabsorptive methods (gastrojejunostomy and bypass).

To date, the only reported treatment for obesity in teenagers or children by endoscopy has used bariatric balloons, which achieved a success of about 10% weight reduction but which, 6 months after removal, invariably ended up with weight gain again. Certainly, endoscopic treatments are likely to offer a non-invasive, reversible “next-step” treatment option, when compared to surgery (103).

### Duodenal Web Division

Congenital duodenal membranes, also known as duodenal webs, are a rare condition with an estimated incidence of 1/10,000–40,000 birth and are often associated with genetic, cardiovascular, or GI abnormalities and are particularly prevalent in syndromes such as Down's or 22q deletion (104). In the case of complete obstruction or atresia, it is usually diagnosed antenatally or soon after birth, but if obstruction is incomplete, diagnosis might be made later in life. Traditionally, treatment was surgical (either laparoscopic or open), but several endoscopic techniques have emerged in the last decade, including endoluminal balloon dilatation, the use of division by sphincterotome, and laser ablation. A combination of endoscopic balloon dilatation and electrocautery endo-knife (MicroKnife, Boston Scientific Microinvasive, Natick, MA, USA)/sphincterotome (Cook MiniTome, Bloomington, IN, USA) has recently been described in 15 children, but this has graduated to balloon dilation only as the use of the endo-knife can be associated with inadvertent perforation of the pancreaticobiliary radicle, which is anatomically opposed to the membrane (104). It is crucial to always check for a secondary, more distal membrane, as this has been observed in up to 20% of cases (104). A single intervention has been sufficient in 60% of the cases, but some of the patients might need a second or third procedure (8). Cases requiring supplementary procedures have been related to the presence of the annular pancreas; hence, Thomson et al. have suggested performing an MRCP prior to the endoscopic procedure—this may highlight the relative position of the ampulla of Vater to the web and suggest whether balloon dilation or balloon and dissection by an endo-knife will be the approach of choice (104).

## The Future

Availability of newer computer chips, better computer processing power with use of 4K and 8K imaging, and improved screen refresh rate are likely to assist the endoscopist in viewing a high-resolution smoothly transitioning dynamic image. Artificial intelligence (computer-assisted diagnosis) with “endoscopists eye tracking” is a technology to further enhance the endoscopist's diagnostic and therapeutic precision. This is also likely to shorten the procedure time with more safety. Unpredictable longer therapeutic procedures can potentially be made safer with the use of CO_2_ insufflation over air insufflation. CO_2_ insufflation is well-tolerated in children and used already in several pediatric GI centers, but a consideration for use in therapeutic pediatric endoscopy with more studies needed to understand its potential benefits has prompted a recent multinational prospective study into its safety and risk mitigation. Robotic-assisted endoscopy is a novel new diagnostic tool for patients who may not tolerate conventional endoscopy, and it may be that therapeutic procedures are possible with this technology in the future. The appropriate application of natural orifice endoluminal surgery (NOTES) in children is yet to be established but maintains a promising future, with incisionless approaches being the eventual aim.

## Data Availability Statement

The original contributions presented in the study are included in the article/supplementary materials, further inquiries can be directed to the corresponding author/s.

## Author Contributions

DS wrote the first draft of the manuscript. NA and MT revised critically the intellectual content. All authors contributed to manuscript revision, read, and approved the submitted version.

## Conflict of Interest

The authors declare that the research was conducted in the absence of any commercial or financial relationships that could be construed as a potential conflict of interest.

## Publisher's Note

All claims expressed in this article are solely those of the authors and do not necessarily represent those of their affiliated organizations, or those of the publisher, the editors and the reviewers. Any product that may be evaluated in this article, or claim that may be made by its manufacturer, is not guaranteed or endorsed by the publisher.
